# Evaluation of the composite materials mixed with calcium phosphate cement and β-tricalcium phosphate granules

**DOI:** 10.1186/s40729-026-00670-w

**Published:** 2026-02-04

**Authors:** Eri Yumoto, Hironori Sakai, Yang Liu, Jianping Liu, Ryo Okaniwa, Ryo Kajihara, Hirokazu Tanaka, Hiroshi Kurita

**Affiliations:** 1 Department of Dentistry and Oral Surgery, Shinshu University School of Medicine, 3-1-1, Asahi, Matsumoto, 390-8621 Japan; 2 Department of Oral and Maxillofacial Surgery, School and Hospital of Stomatology, Hebei Medical University, Hebei Technology Innovation Center of Oral Health, Hebei Province, Shijiazhuang, 050017 China

**Keywords:** CPC, β-TCP, Artificial bone, Osteoconductive, Biodegradation

## Abstract

**Purpose:**

Calcium phosphate cement (CPC) demonstrates excellent shape retention; however, the material exhibits a prolonged resorption period. Despite its inferior mechanical strength and poor shape retention, beta-tricalcium phosphate (β-TCP) offers excellent osteoconductivity. We previously reported that incorporating β-TCP into CPC enhances mechanical strength and shape retention. However, the prolonged setting time poses a challenge for clinical application. We aimed to improve the clinical usefulness of the CPC/β-TCP composite material.

**Materials and methods:**

Samples with different mixing ratios of β-TCP (SUPERPORE) to quick-setting type of CPC (BIOPEX^®^-R Quick Type) (C0, C10, C30, and C50 with mixing ratios of β-TCP of 0, 10, 30, and 50 wt%, respectively) were prepared. We evaluated the material properties. New bone formation was evaluated by histological and histopathological analysis after 4 and 8 weeks of implantation into the calvarial bone of Wistar rats.

**Results:**

This material was set in approximately 4 min. As the β-TCP content increased, the compressive strength decreased significantly. The average compressive strength C30 was 1.5 MPa. Penetration tests confirmed good permeability for C30 and C50. In the histological study, better new bone formation was observed in the C30 and C50 at 4 and 8 weeks.

**Conclusion:**

These results suggested that the composite material made in this study had a shorter setting time and improved penetration than that in the previous study and confirmed its biocompatibility and osteoconductivity. Its compressive strength was low; therefore, it is necessary to examine whether the material can withstand the vertical and horizontal augmentation of the jawbone.

## Introduction

In the oral and maxillofacial region, bone defects can be caused by tumors, jaw cysts and other diseases, as well as by treatment such as extirpation or removal of the disease. Furthermore, alveolar bone defects are caused by dental diseases (the progression of dental caries or periodontal disease) [1–3]. Repairing bone defects is necessary for restoring functionality and aesthetics in the oral and maxillofacial regions. Autografts are the gold standard treatment for bone augmentation [1–9]. Autogenous bone grafts have the biological properties of osteoconduction, osteoinduction, and osteogenicity [3, 10]. However, autogenous bone grafts are associated with morbidity at the donor site and surgical risks such as bleeding, inflammation, chronic pain, and limited harvestability [1–3, 5, 8]. Therefore, various artificial bones have been developed as alternatives. It is preferable for artificial bone used in the oral and maxillofacial region to be quickly absorbed and replaced by new bone because there is a high possibility of infection caused by oral bacteria, if artificial bone is left in place for a long period of time. Furthermore, bone defects in the oral and maxillofacial regions are often complex in shape. Sufficient vertical height and width of the alveolar ridge are necessary for functional and aesthetic reconstruction, including dental implant treatment [1, 8, 11]. Granular and block-shaped bone fillers may not fill complex bone defects and give them the desired shape. Therefore, we aimed to develop a new bone filler of sufficient strength, good osteoconductivity, and rapid biodegradability for use in dental implant treatment, periodontal surgery, cyst cavity, and extraction sockets. We created a composite material by mixing two types of materials: calcium phosphate cement (CPC), which has paste-type formability, and β-tricalcium phosphate (β-TCP), which is granular and porous. Although β-TCP has weak mechanical properties, it has good biocompatibility, osteoconductivity, and bioresorbability [9, 12–19]. CPC exhibits osteoconductivity, biocompatibility and moldability. However, it is poorly absorbed, and biodegradation is slow because it precipitates as hydroxyapatite (HA) in vivo [2, 20–24]. In our previous study, a composite created by adding an equal weight percentage of porous β-TCP (Cerabeta^®^; NGK Spark Plug Co. Ltd, Aichi, Japan) to CPC (Cerapaste^®^; NGK Spark Plug Co. Ltd, Aichi, Japan) was implanted into the femur of a rabbit. The material properties were evaluated in vitro. The results of the study suggested that the composite (CPC: β-TCP = 50: 50) had good biodegradability and mechanical strength compared with those of CPC alone [24]. However, the composite posed problems of long hardening time; thus, its use was deemed difficult for clinical use. Therefore, in this study, we evaluated the material properties of a new product in vitro. In addition, biodegradation and osteoconductivity were evaluated in vivo by implantation in the calvarial bone of Wistar rats, which has the same pattern of bone formation as the jawbone. We developed a new CPC/β-TCP composite material that has a faster setting time.

## Materials and methods

### CPC and β-TCP

Two new commercially available artificial bone materials were used in this study. The CPC used in this study was BIOPEX^®^-R Quick Type (HOYA Technosurgical Corporation, Tokyo, Japan). It consists of a mixture of powder and liquid. The powder mainly consists of α-tricalcium phosphate (α-TCP) with other components including tetracalcium phosphate (TTCP), calcium hydrogen phosphate (DCPA), hydroxyapatite and magnesium phosphate. The liquid is mainly composed of sodium chondroitin sulfate, disodium succinate anhydrous, sodium hydrogen sulfite, and water. The β-TCP used in this study was SUPERPORE (HOYA Technosurgical Corporation, Tokyo, Japan), which consists of granules with a diameter of 0.6–1.0 mm. The granules are characterized by a hollow interior and porous structure. The porosity is 75% (± 3%), the macroporosity is 50–300 μm or greater, and the microporosity is 10 μm or less. The communication hole is 50 μm or greater. CPC and β-TCP were kindly provided by HOYA Technosurgical Corporation.

### Preparation of the study samples (CPC/β-TCP composite granules with different ratios)

Test materials with different blending ratios of β-TCP to CPC (C0, C10, C30 and C50, with blending ratios of β-TCP of 0, 10, 30 and 50 wt%, respectively) were prepared in a room maintained at controlled temperature (25  ±  3℃) and humidity (50  ± 10%). First, the CPC powder was kneaded with the malaxation liquid. When the CPC became clayey, different wt% of β-TCP granules were added to the CPC paste and softly mixed. The CPC powder/mixing liquid ratio (P/L) was 3.03 for C0, 3.03 for C10, 3.23 for C30, and 1.96 for C50. The mixing ratios were determined with reference to previous studies to identify a composition that provides a balance between bone formation and mechanical strength. C10 was included as a low β-TCP content group for comparison with higher β-TCP-containing composites. In addition, at β-TCP contents above 50 wt%, the material failed to form a coherent and stable composite with CPC.

### In vitro study

#### Preparation of composite materials

**Fig. 1:**
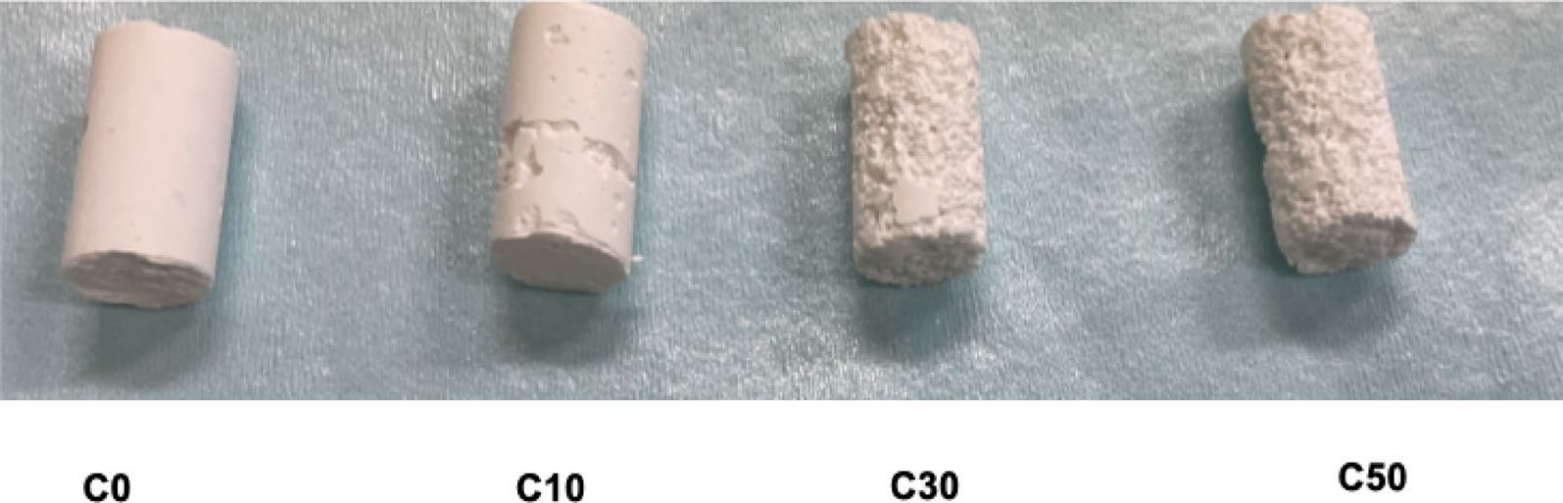
Composite of CPC and β-TCP

The mixed composite materials were filled into plastic cylindrical containers (internal diameter: 6.5 mm, height: 10 mm). After hardening, the composites were removed from the container to prepare the experimental samples (Fig. 1).

#### Measurement of paste setting time

The setting time was measured using a method based on the principles of ISO 9597 and ISO 9917–1, according to the procedure reported in a previous study [24]. A flat-tipped 2-mm-diameter needle was gently placed on the top surface of the hardened body at each time point. The setting time was defined as the time at which no indent was formed by the needle (300 g in weight). Measurements were carried out 5 times and the values were averaged.

#### Measurement of the compressive strength of the hardened body

The compressive strength was measured according to the procedure outlined in a previous study [24], based on the principles of ISO 13175-3. After setting, the hardened body was immediately removed, immersed in distilled water, incubated at 37 ± 1 °C for 24 h, and subsequently used for measurement of compressive strength. Compressive strength was measured using a universal testing machine (INSTRON 5982^®^, Instronjapan Corporation, Kanagawa, Japan) with a crosshead speed of 0.5 mm/min, and the mean value of 5 samples (n = 5) was calculated.

#### Density and porosity 

The porosity of the bone substitutes was evaluated using the following formula. To estimate the bulk density and total porosity of the hardened body, samples were prepared in the same way as for measuring the compressive strength. The total porosity (P) of the specimens was calculated as follows: the dry weight (M) of each specimen was divided by the volume (V) to obtain the density (D). This value was divided by the theoretical density of hydroxyapatite (DHAp = 3.16 g/cm^3^), subtracted from 1, and expressed as a percentage.

$$ \begin{aligned} {\text{D }} = & {\mathrm{M}}/{\mathrm{V}} \\ {\text{P }} = & { 1}00 \times \left( {{1} - {\mathrm{D}}/{\mathrm{DHAp}}} \right) \\ \end{aligned} $$

       D = Density.

M = Dry weight.

V = Volume.

P = Porosity.

DHAp = Density of hydroxyapatite = 3.16 g/cm^3^

#### SEM micrographs of the cross-section of the hardened body

The surface of the hardened body was analyzed via scanning electron microscopy (SEM, JSM -6510LV).

#### X-ray computed microtomography of the hardened body

The hardened body was scanned using 3D micro-CT (Latheta, LCT-200, Hitachi, Ltd, Tokyo, Japan). The X-ray source operated at 50 kV and 0.5 mA, a scan time of 21.2 × Ns/rotation, and a voxel size of 24 × 48 μm.

#### Penetration test

Each hardened body was immersed in a red propylene glycol solution for 20 min and for 24 h, after which the hardened body was cut into a cross section in the center, and the red-stained area was measured using NIH ImageJ software (National Institutes of Health, Bethesda, MD). The average of 5 samples was calculated. The penetration rate (Pr) was calculated according to the following formula:

$$ {\text{Pr }} = {\text{ red stained area}}/{\text{total area }} \times { 1}00\% $$

### In vivo study

#### Animal experiments

**Fig. 2:**
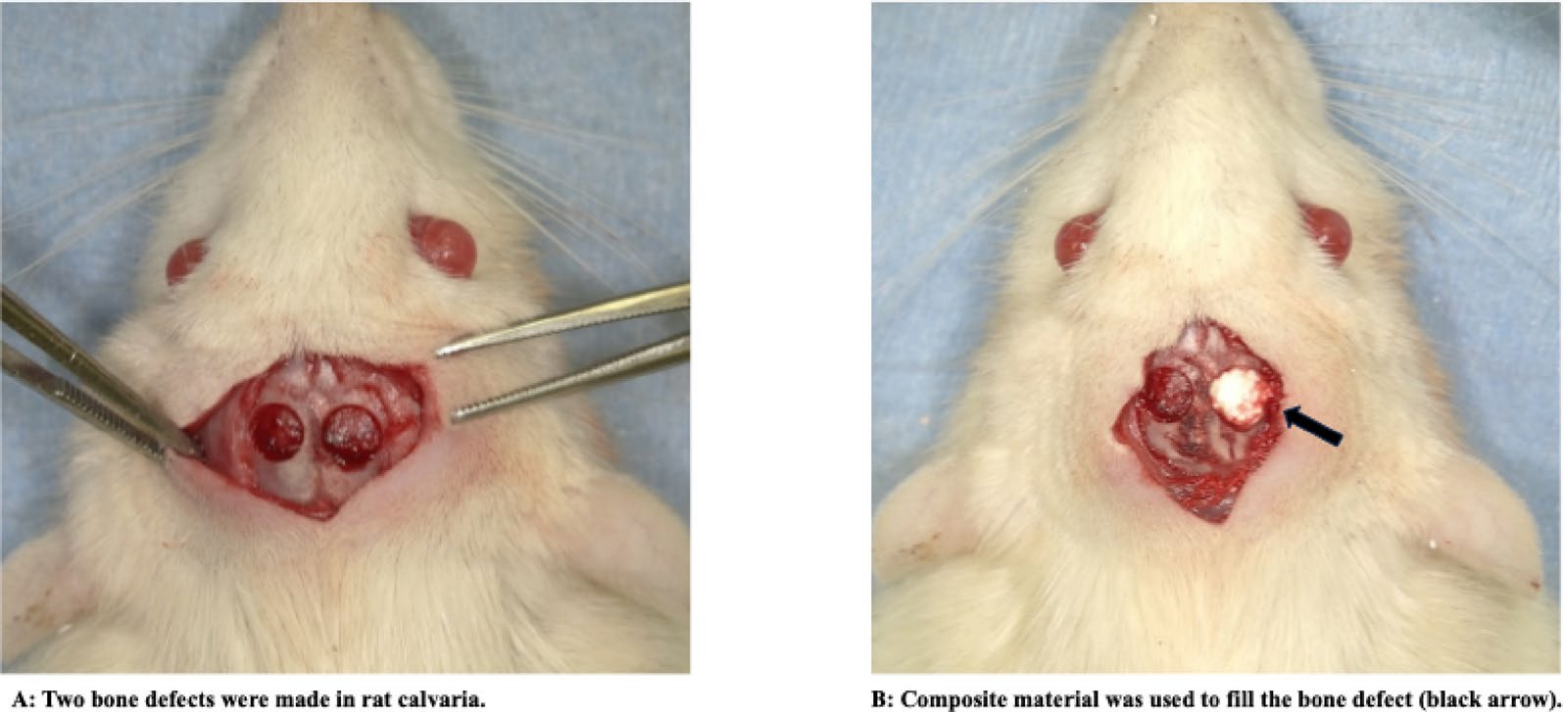
In vivo study

The animal protocols were reviewed and approved by the ethical committee of Shinshu University (Approval number 024042). Wistar rats (male, 10 weeks of age) were purchased from SLC (Shizuoka, Japan) and kept in a controlled environment. After being premedicated with isoflurane ether via inhalation, the animals were anesthetized with a mixture of dexmedetomidine hydrochloride (0.375 mg/kg), midazolam (2 mg/kg), and butorphanol tartrate (2.5 mg/kg) via subcutaneous injection. The head of each rat was incised with surgical scissors, and the flap was formed under the subperiosteal space and lifted to expose the calvaria. Two bone defects of 4.5 mm in diameter were made in the calvaria using a trephine bar, φ4.5 mm (Fig. 2A). Composite materials were made as test materials with different blending ratios of β-TCP to CPC (C0, C10, C30 and C50, with blending ratios of β-TCP of 0, 10, 30 and 50 wt%, respectively). These materials were gently inserted into the bone defects. No material was implanted in the control group (Fig. 2B). Once the defects had been filled, the periosteum was sutured using absorbable sutures, and the skin flap was sutured using silk sutures. Wistar rats were sacrificed using carbon dioxide gas four and eight weeks after sample implantation. Each group consisted of five samples(n = 5). This number was determined based on the principles of the 3Rs (Replacement, Reduction, and Refinement) in animal experimentation, while ensuring the minimum number of samples required for valid statistical analysis.

#### Histological analysis

Calvaria were harvested as specimens and fixed in 4% paraformaldehyde phosphate buffer solution. Fixed specimens were decalcified using K-CX^®^ (Falma, Tokyo, Japan). Bone samples were embedded in paraffin, and sectioned. All sections were subsequently stained with hematoxylin/eosin. Bone samples were observed using a light microscope (BZ9000, All-in-One Fluorescence Microscope, Osaka, Japan; Vectra3 image system, Akoya Biosciences, Marlborough, MA). The area of newly formed bone and the combined area of remaining material and void spaces were measured using NIH ImageJ software (National Institutes of Health, Bethesda, MD). Histomorphometric analysis was performed by three investigators, and two investigators were blinded with respect to group allocation. The areas were expressed as percentages of the total area, and the values represent the mean of measurements obtained by the three investigators. Newly formed bone area was measured in the C0, C10, C30, C50 and control groups; the combined area of remaining material and void spaces was measured in the C0, C10, C30 and C50 groups.

### Statistical analysis

Mean values and standard deviations were calculated using Microsoft Excel software. Statistical analyses were performed using SPSS version 31.0. The Mann–Whitney U test was used to compare the two experimental time points within each group, while the Kruskal–Wallis test followed by Dunn’s post hoc test was used to compare differences among the five groups at the same time point. A P value of < 0.05 was considered statistically significant.

## Results

### In vitro study

#### Setting time

The setting time of the CPC (C0) was 217 ± 22.5 s. The setting time with additional β-TCP was 217 ± 35.8 s for C10, 216 ± 8.2 s for C30, and 290 ± 46.4 s for C50. There was no difference in the setting time between C0, C10, and C30. However, C50 had a longer setting time than the other groups. Furthermore, the setting time was significantly shorter in each group in the present study than in our previous study (Table 1).

**Table 1:** Setting time of the CPC/β-TCP composite material (n = 5)

	Mean ± SD (seconds)	
	Present study	Reference (results from a previous study [24])*
C0	217 ± 22.5	600 ± 65.4
C10	217 ± 35.8	–
C30	216 ± 8.2	900 ± 103.2
C50	290 ± 46.4	1080 ± 84.6

SD, standard deviation

*CPC (Cerapaste^®^; NGK Spark Plug Co. Ltd, Aichi, Japan) and β-TCP (Cerabeta^®^ granules; NGK Spark Plug Co. Ltd, Aichi, Japan) were used

#### Compressive strength

The mean compressive strengths of C0, C10, and C30 were 21.6, 4.9, and 1.5 MPa, respectively. As β-TCP content increased, the compressive strength significantly decreased. A significant difference was observed among these mixing ratios (Table 2; Kruskal–Wallis test, *P* < 0.05). C50 was too fragile to measure.

**Table 2:** Compressive strength of hardened composites of CPC cement and β-TCP granules (n = 5)

Mean ± SD (Mpa)			*P*-value
C0	C10	C30	
21.6 ± 2.50	4.9 ± 1.47	1.5 ± 0.47	0.002

#### Total porosity rate

The total porosity rates of C0, C10, C30, and C50 are shown in Table 3. The average porosity rate of C0, C10, C30, and C50 were 31%, 38%, 45%, and 68%, respectively. Porosity rates increased with increasing β-TCP content. A significant difference in the porosity rate was observed among these mixing ratios (Kruskal–Wallis test, *P* < 0.05).

**Table 3:** Total porosity of hardened composites of CPC cement and β-TCP granules (n = 5)

Mean ± SD (%)				*P*-value
C0	C10	C30	C50	
31.4 ± 1.47	38.3 ± 0.71	45.5 ± 2.51	68.2 ± 4.87	*P* < 0.001

#### SEM micrograph and micro-computed tomography (micro-CT)

**Fig. 3:**
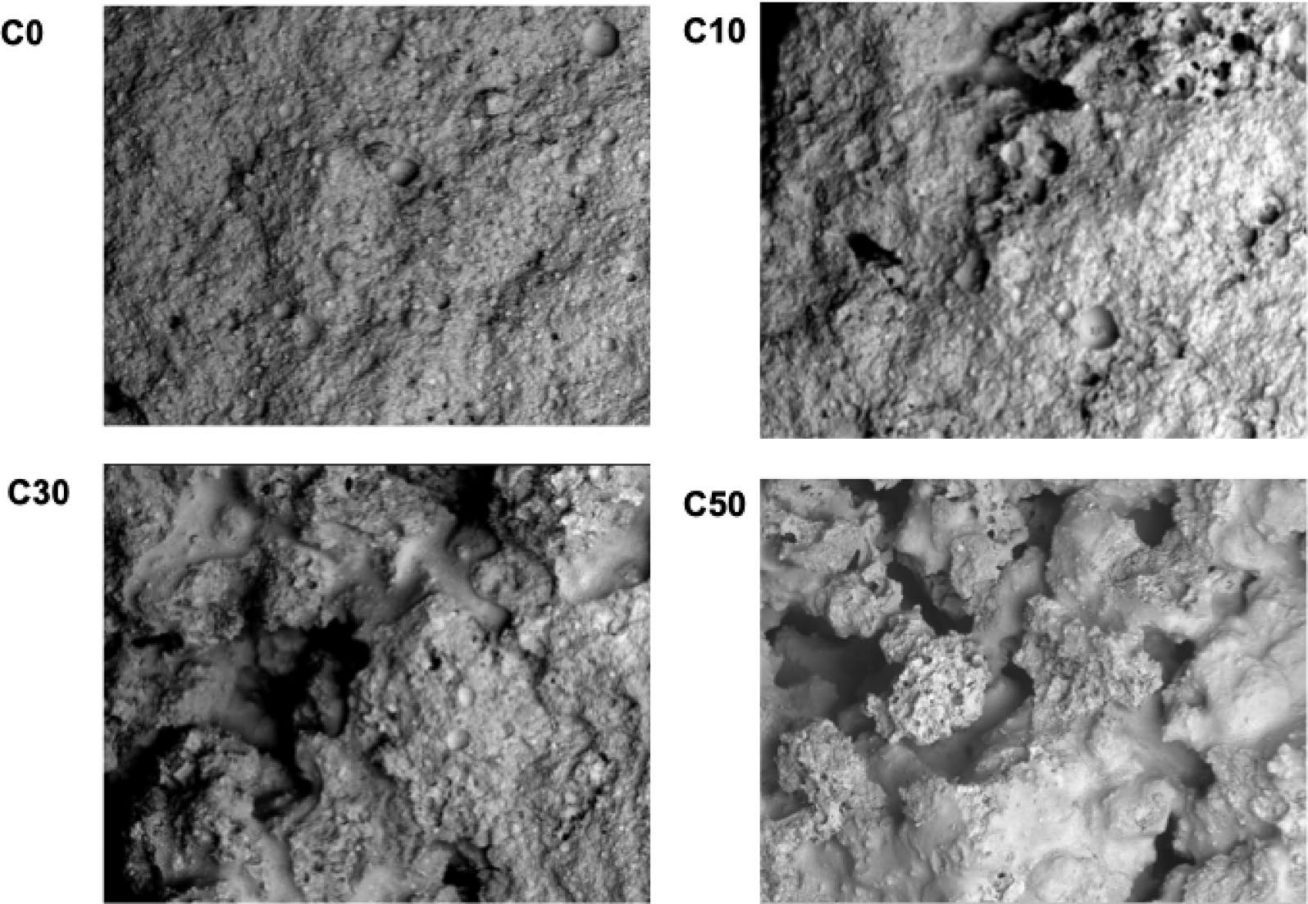
Surface morphology of the cross-section of hardened composite as observed by SEM

**Fig. 4:**
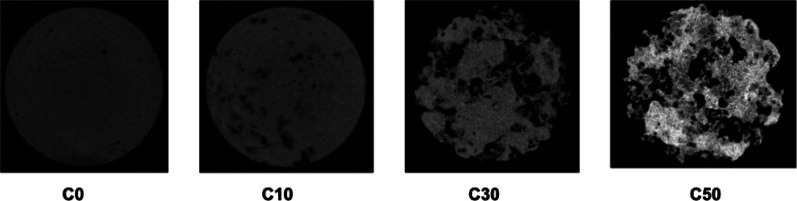
Surface morphology of the cross-section of hardened composite as observed by micro-CT

The surface morphology of the cross-section of the hardened composite was observed by SEM and micro-CT (Fig. 3 and 4). No pores were observed in C0. Pores were observed in the cross section of the hardened bodies of C10, C30, and C50. CPC cement was present between the β-TCP granules and adhered to them. The pores of the body of the complex were not filled with CPC. Micropores and macropores were observed in β-TCP, and the number of pores in the samples also increased with increasing β-TCP content.

#### Penetration test

**Fig. 5:**
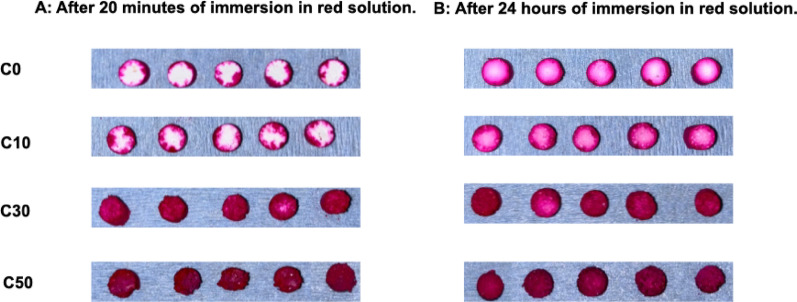
Cross sections of the hardened body. The cross sections of the hardened body were observed to determine the penetration rate. For C30 and C50, the dye solution was reached the center of composites even after 20 min, and the in interior was deeply stained

To examine the penetration rate, cross sections of the hardened bodies were observed after 20 min and 24 h immersion in red dye solution (Fig. 5). In the C0 samples, unstained areas were present at both 20 min and 24 h, and no red staining was observed within the interior of the hardened composite cross-section. In the C10 samples, unstained areas were observed only after 20 min. In both C30 and C50 samples, the red dye solution reached the center of the hardened body at all immersion times (Table 4). These results indicate that the hardened material containing β-TCP demonstrates superior permeability.

**Table 4:** Stained area of composite cross sections after 20 min and 24 h (n = 5)

	Mean ± SD (%)	
	At 20 min	At 24 h
C0	45.99 ± 3.12	98.56 ± 1.77
C10	67.26 ± 6.57	100
C30	100	100
C50	100	100

### In vivo study

**Fig. 6:**
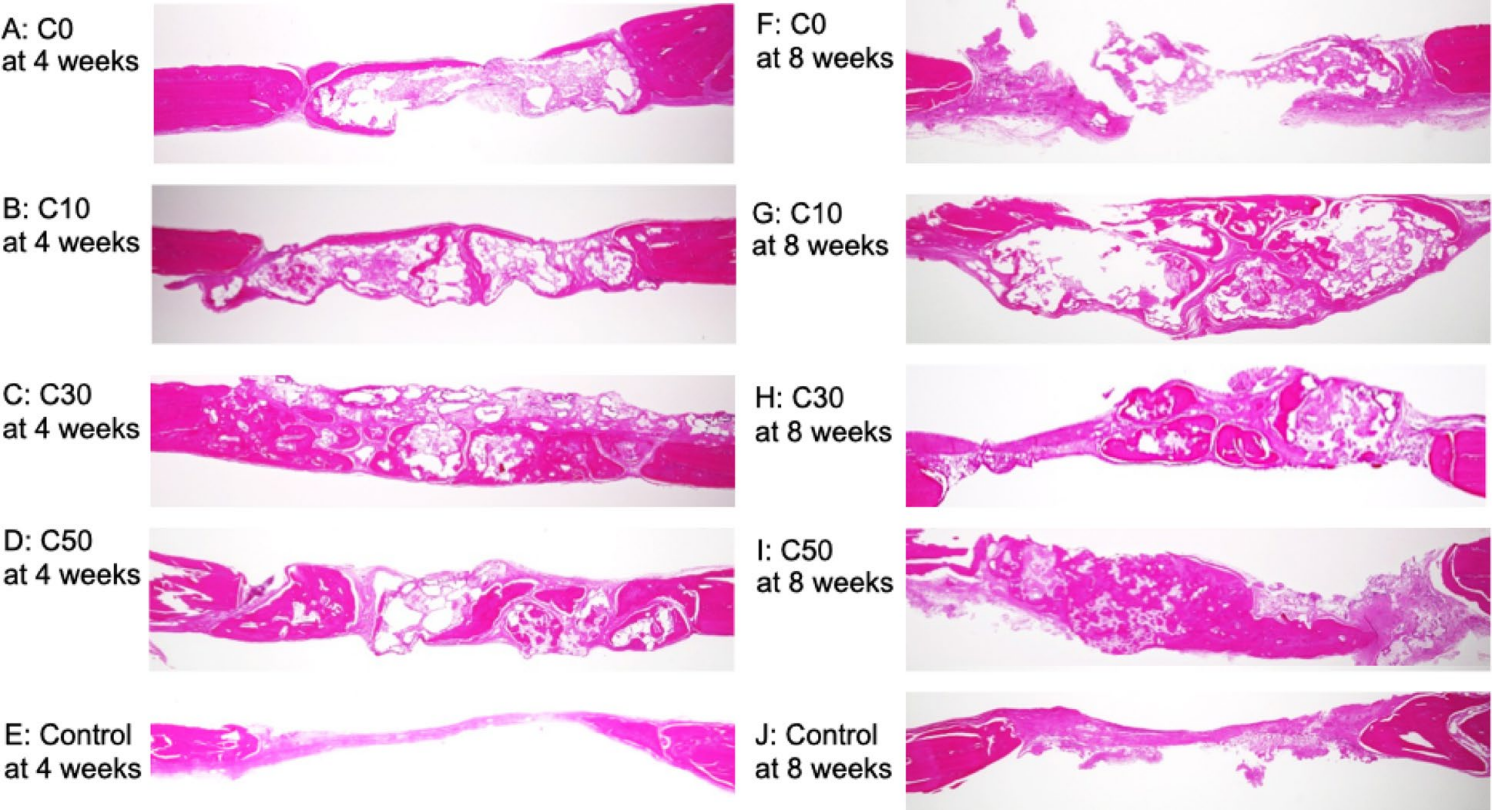
Histological images of specimens of different composites of CPC and β-TCP (C0, C10, C30 and C50) and the control at 4 (**A**–**E**) and 8 weeks (**F**–**J**) (hematoxylin and eosin stain × 2). **A** C0 at 4 weeks **B** C10 at 4 weeks **C** C30 at 4 weeks **D** C50 at 4 weeks **E** Control at 4 weeks **F** C0 at 8 weeks **G** C10 at 8 weeks **H** C30 at 8 weeks **I** C50 at 8 weeks J Control at 8 weeks. In composites of CPC and β-TCP (C30 and C50) in rats euthanized at 4 and 8 weeks postoperatively (**C**, **D** and **H**, **I**), good new bone formation was observed in the central area

**Fig. 7:**
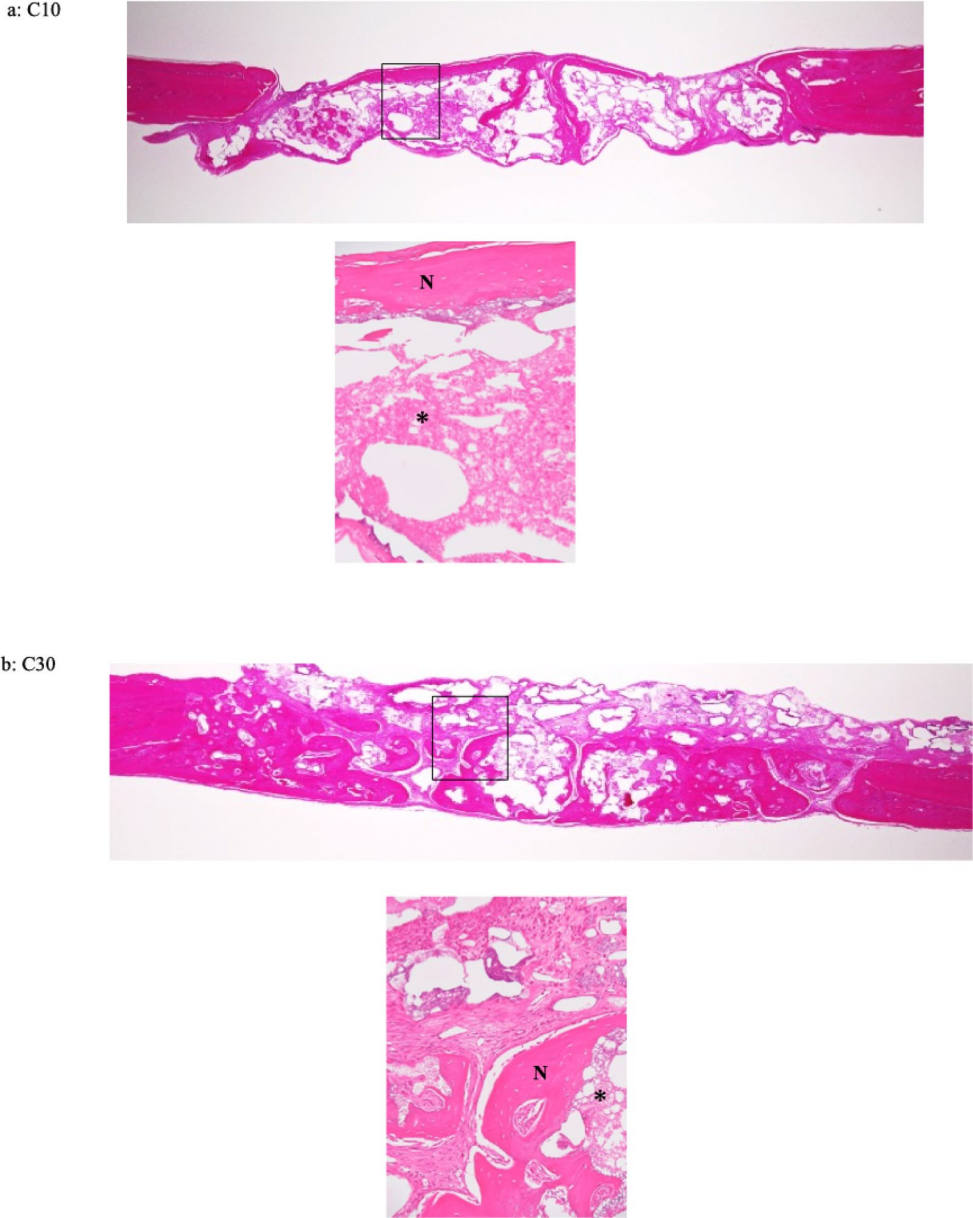

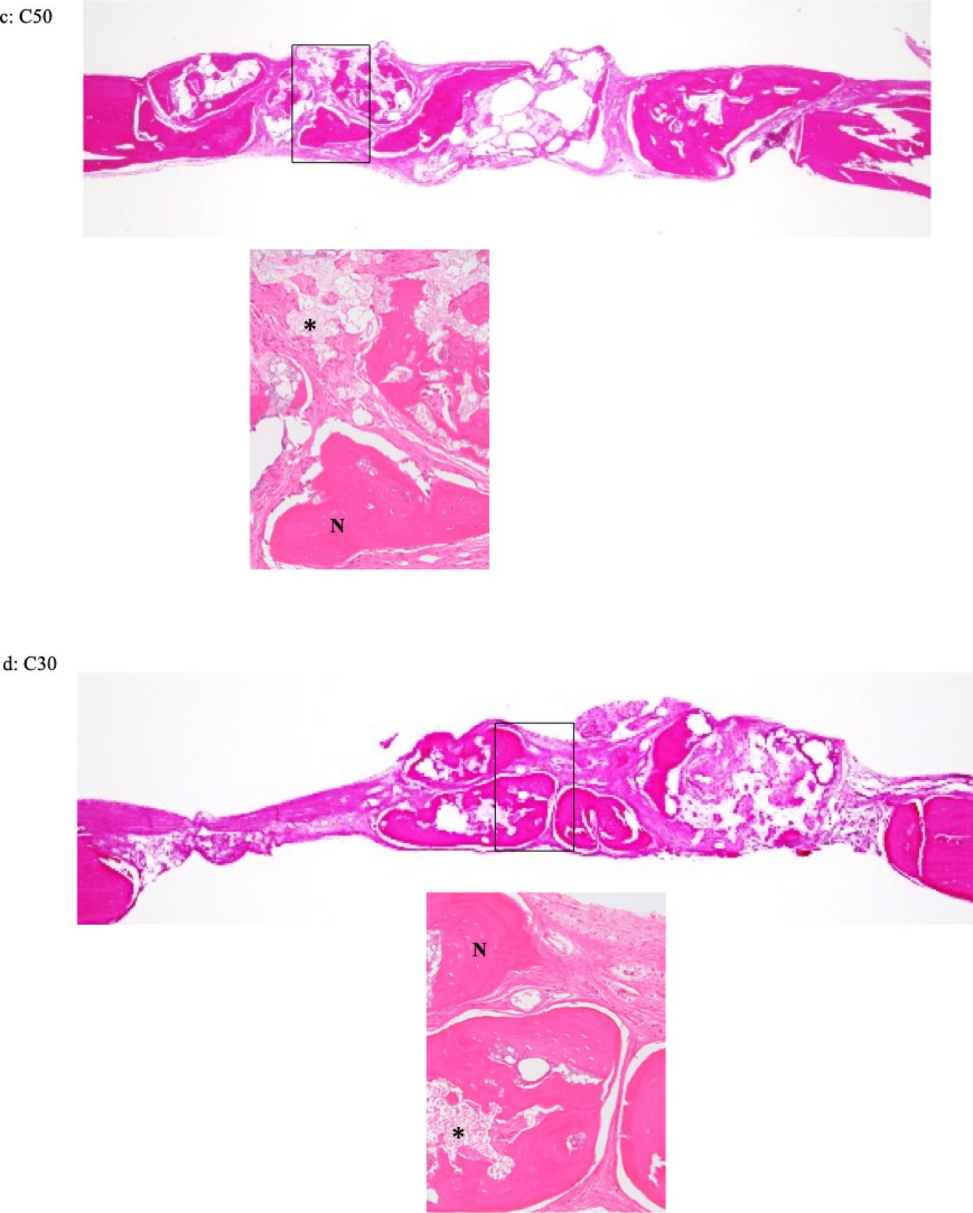

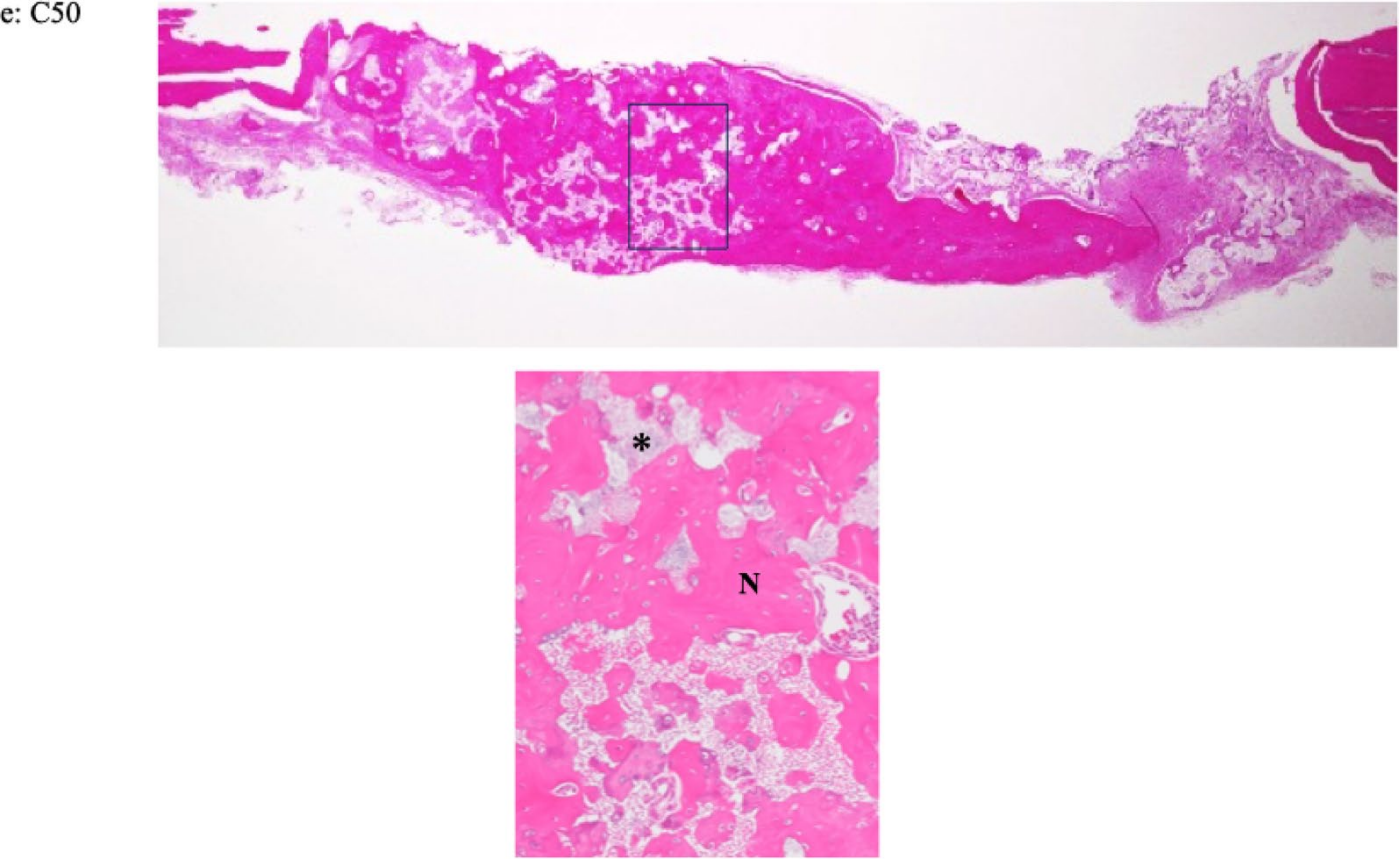
Histological images of specimens of different composites of CPC and β-TCP (C10, C30 and C50) at 4 (**a**–**c**) and 8 weeks (**d**, **e**) (hematoxylin and eosin stain × 80). 'N' and an asterisk (*) indicate new bone formation and material, respectively. **a** C10; New bone formation was observed beneath the periosteum. Remaining material was present in direct contact with the new bone formation. No marked capillary proliferation or inflammatory cell infiltration was observed. **b** C30; New bone formation was observed in contact with the remaining material. Granulation tissue containing inflammatory cells, capillaries, and fibroblasts was present around the new bone formation. The granulation tissue was also in direct contact with the remaining material. **c** C50; New bone formation and fibrous connective tissue were observed in contact with the remaining material. Remaining material was continuously present within the new bone formation. **d** C30; New bone formation was observed in continuity with fibrous connective tissue. Remaining material was present within and in contact with the new bone formation. **e** C50; New bone formation and remaining material were intermingled, and new bone formation was observed in continuity with the remaining material. No apparent fibrous connective tissue was observed

Histological images of each specimen are shown in Fig. 6. In the C30 and C50 groups, new bone formation was predominantly observed in the central region of the defect at both 4 and 8 weeks after implantation. In contrast, in the C0 and C10 groups, new bone formation was limited and mainly observed around the periosteum and adjacent to the material at both time points. In the control group, only a small amount of new bone was observed at the margins of the pre-existing bone, and new bone formation was rarely detected at 4 and 8 weeks after implantation. At both 4 and 8 weeks, remaining material was observed in all material-implanted groups (Fig. 6). At 4 weeks, vascularized granulation tissue was observed adjacent to the newly formed bone in the C30 group. In contrast, the C50 group showed fewer capillaries than the C30 group and exhibited fibrous connective tissue. At 8 weeks, fibrous connective tissue was observed near the newly formed bone and the remaining material in the C30 group, whereas no apparent fibrous connective tissue was observed in the C50 group (Fig. 7). New bone formation was compared by the area ratio. At 4 weeks after implantation, the mean values of bone formation were 5.22 ± 4.21% for C0, 7.39 ± 7.12% for C10, 25.92 ± 7.17% for C30, 13.23 ± 7.92% for C50, and 8.12 ± 12.39% for the control. At 8 weeks after implantation, the mean values of bone formation were 4.73 ± 3.58% for C0, 6.17 ± 3.95% for C10, 14.43 ± 6.25% for C30, 18.96 ± 7.57% for C50, and 2.35 ± 4.70% for the control (Table 5). There was a significant difference in bone formation among the groups at both 4 and 8 weeks (Kruskal–Wallis test, *P* < 0.05). At 4 weeks, the C30 group showed significantly greater bone formation compared with the C0, C10, and control groups. At 8 weeks, the C30 group showed significantly greater bone formation compared with the control group, while the C50 group exhibited significantly higher bone formation than the C0, C10, and control groups (Dunn’s post hoc test *P* < 0.05). No statistically significant differences in bone formation were observed between 4 and 8 weeks in any group (Mann–Whitney U test, *P* > 0.05). The ratios of the combined area of remaining material and void spaces are shown in Table 6. Significant differences in the combined area ratios were observed among the groups at both 4 and 8 weeks (Kruskal–Wallis, * P* < 0.05). In addition, a statistically significant difference in the combined area ratio between 4 and 8 weeks was observed in the C30 group (Mann–Whitney U test, *P* < 0.05), whereas no significant differences were detected in the other groups (Mann–Whitney U test, *P* > 0.05).

**Table 5:** Mean and standard deviation of the percentage of new bone formation by group and time of evaluation (n = 5)

	C0	C10	C30	C50	Control
	Mean ± SD	Mean ± SD	Mean ± SD	Mean ± SD	Mean ± SD
At 4 weeks (%)	5.22 ± 4.21	7.39 ± 7.12	25.92 ± 7.17	13.23 ± 7.92	8.12 ± 12.39
At 8 weeks (%)	4.73 ± 3.58	6.17 ± 3.95	14.43 ± 6.25	18.96 ± 7.57	2.35 ± 4.70

**Table 6:** Mean and standard deviation of the percentage of combined area of remaining material and void spaces by group and time of evaluation (n = 5)

	C0	C10	C30	C50
	Mean ± SD	Mean ± SD	Mean ± SD	Mean ± SD
At 4 weeks (%)	54.73 ± 11.72	44.26 ± 11.74	31.23 ± 8.70	9.79 ± 6.68
At 8 weeks (%)	38.52 ± 6.36	43.73 ± 3.60	9.67 ± 7.90	3.23 ± 3.20

Significant differences were observed at both 4 and 8 weeks. At 4 weeks, the C50 group showed a significantly lower area ratio compared with the C0 and C10 groups. At 8 weeks, the C30 group showed a significantly lower area ratio compared with the C10 group, while the C50 group exhibited significantly lower area ratios compared with the C0 and C10 groups (Dunn’s post hoc test, *P* < 0.05)

## Discussion

In oral and maxillofacial surgery, artificial bone grafts are mainly used for dental implant treatments and are generally granular [9]. However, granular bone grafts do not retain their shape, so they are combined with membranes and bone tacks. This makes the procedure cumbersome and can result in the granules moving and spilling out. In addition, horizontal and vertical bone grafts are difficult to perform, and the graft is vulnerable to shocks when eating. It is necessary to rest for long periods until the graft stabilizes [25]. Therefore, the artificial bone used in oral and maxillofacial surgery needs to be formable and mechanically strong. In addition, the use of artificial materials in the oral cavity carries a high risk of infection; the materials must be quickly absorbed and replaced by bone tissue. We focused on two artificial bone materials. When powder and liquid CPC are mixed, the mixture becomes clay-like and can be molded into any desired shape [2, 20, 22]. However, CPC does not have macropores and precipitates as HA, resulting in slow biodegradation [2, 22–24]. Accordingly, we created a composite material combining CPC and β-TCP, which exhibited excellent bioabsorbability. In a previous study, we created a CPC (Cerapaste^®^) and β-TCP (Cerabeta^®^) composite material and evaluated its properties in vitro. The CPC used consists of mixed powder and liquid. The powder contains tetracalcium phosphate (TeCP: 67.5 wt %) and dibasic calcium phosphate anhydrous (DCPA: 32.5%), and the liquid contains water and dextran sulfate sodium [24]. We also evaluated its biodegradability and osteoconductivity in vivo by implanting it in the femurs of New Zealand white rabbits. We confirmed that adding an equal wt% of CPC and β-TCP to the composite material resulted in good biodegradability and sufficient mechanical strength. However, the setting time was long at 18 min [24]. Due to the extended treatment duration and associated patient burden, the composite material was deemed unsuitable for clinical use.

In this study, in order to shorten the setting time, we switched to a fast-setting CPC material (BIOPEX^®^-R Quick Type). Although the previously used CPC (Cerapaste^®^) typically required 5–20 minutes to set, Biopex^®^-R reportedly sets in approximately 4 minutes according to manufacturer information. In this study, the setting times for C0, C10 and C30 were just under 4 min, whereas C50 had the longest setting time at just under 5 min. This was a significant reduction in setting time compared with that reported in the previous study. There is enough time for operation, and it was confirmed that the setting times in each composite were suitable for clinical use. It is well established that cement setting time can be modulated by changing powder particle size, adjusting the liquid-to-powder ratio, or incorporating commonly available calcium and/or phosphate ions, crystal nuclei, or crystal growth inhibitors. [26]. In this study, the setting time was shortened because the product used contained DCPA particles in a smaller size range, and the powder components and proportions were different from those used in the previous study. C50 required a longer setting time than the other composite materials because more liquid was needed to fabricate it. This material is intended for use in load-bearing areas, and therefore, it is necessary to evaluate its mechanical strength. Compared with that reported in a previous study, the compressive strength of both the CPC sample alone and the sample mixed with β-TCP decreased. The compressive strength of cortical bone ranges from 90–230 MPa, whereas that of trabecular bone ranges from 2–45 MPa [26]. In this study, the compressive strengths of C0 and C10 were similar to that of the trabecular bone, whereas that of C30 was lower. The compressive strength of this material was lower than that of the materials used in previous studies. High L/P ratios lead to increased injectability but decreased mechanical properties [26]. In this study, the CPC powder/mixing liquid ratio (P/L) was 3.03 for C0 and C10, 3.23 for C30 and 1.96 for C50. In the previous study, the P/L ratio was 3.57 for C0, 3.18 for C30 and 2.1 for C50. The increased liquid volume is likely to be one factor contributing to the decrease in compressive strength. The compressive strength also decreases with increasing porosity [27]. The porosity of the β-TCP granules (Cerabeta^®^) used in the previous study was 35%, with a macroporosity of 200 µm and microporosity of 1 µm, while the porosity of the β-TCP granules (SUPERPORE) used in this study was significantly greater at 75%, with a macroporosity of 50–300 µm or greater and microporosity of 10 µm or less. This characteristic is thought to have caused a decrease in the mechanical properties of the material.

The increased porosity also resulted in a significantly improved penetration rate. The results of the previous study confirmed that the red dye solution had reached the center of the composite material for C50 after 24 h of immersion. In this study, it was confirmed that the red dye solution had reached the center of the composite material for C30 and C50 after 20 min of immersion. The composite material in this study demonstrated excellent porosity and permeability. Good porosity and permeability are essential requirements for artificial bone. Porosity enables cells such as osteoblasts and mesenchymal cells to migrate and proliferate and allows capillaries to form and the matrix to be deposited into the pores. This contributes to the rapid formation of new bone [2, 20, 28–34]. Another important aspect of porosity is the interconnected structure. This provides channels for cells, blood and nutrients to penetrate the interior, thereby promoting new bone formation and biodegradation [22, 33, 34]. The pores in the CPC/β-TCP composite did not disappear when the two materials were mixed, and an interconnected structure was present. In general, the first 8 weeks are regarded as the early healing phase of jawbone regeneration. Recent clinical reviews on implant placement timing also describe the postoperative 4–8-week interval as an early healing period relevant to bone regeneration and implant therapy [35, 36].

Accordingly, the present study evaluated new bone formation induced by the composite materials at 4 and 8 weeks after implantation, focusing on this early healing phase. In the present study, at both 4 and 8 weeks after implantation, new bone formation in the central region of the defect was observed in C30 and C50 compared with C0, C10, and the control group. These findings suggest that materials with a higher β-TCP content (C30 and C50) may have provided a more favorable environment for bone regeneration up to 8 weeks after implantation. In addition, the newly formed bone observed in the central region of the defect was in contact with the remaining material as well as with the surrounding granulation tissue or fibrous connective tissue, indicating that bone formation progressed using the implanted material as a scaffold. Although histomorphometric analysis revealed no statistically significant differences in the amount of new bone formation between 4 and 8 weeks in any group, histological observations demonstrated differences in the progression of bone formation depending on the observation timing and material composition. While histomorphometric analysis quantitatively evaluates the amount of newly formed bone, histological observation allows qualitative assessment of tissue characteristics, such as vascularization, connective tissue response, and the maturation stage of bone. At 4 weeks, newly formed bone and the associated responses of the surrounding connective tissue were clearly observed. In particular, vascularized granulation tissue was present adjacent to the newly formed bone in C30, whereas reduced vascularity and the presence of fibrous connective tissue were observed in C50, suggesting that bone formation in C50 had progressed to a more advanced stage at this time point. At 8 weeks, further maturation of the newly formed bone was histologically confirmed. In C30, fibrous connective tissue was still observed near the newly formed bone and remaining material, suggesting ongoing tissue remodeling. In contrast, fibrous connective tissue was rarely observed in C50, indicating a more advanced stage of bone maturation. In the present study, partial decalcification of the remaining material occurred during histological specimen preparation. Therefore, the remaining material and void spaces were evaluated as a combined area. Histomorphometric analysis showed that area ratios of the combined remaining material and void spaces tended to decrease with increasing β-TCP content. When comparing the results between 4 and 8 weeks, a statistically significant difference was observed only in C30, indicating that material resorption progressed from 4 to 8 weeks in this composition. In contrast, in C50, a large portion of the material may have already been resorbed by 4 weeks, which could explain the absence of a significant difference between the two time points. In vivo, as the CPC reaction proceeds, HA is precipitated [2, 20–24]. β-TCP is generally considered to be resorbed more rapidly than HA [16, 18].

Previous studies using X-ray diffraction analysis have demonstrated that an increase in β-TCP content is associated with a decrease in HA [24]. In the present study, it is therefore likely that a substantial amount of HA precipitated from CPC remained within the defect. Histological observations indicated that all CPC/β-TCP composite materials (C10, C30, and C50) remained unabsorbed up to 8 weeks after implantation. While rapid degradation of the material is necessary to prevent infection, excessively fast degradation may cause the scaffold to disappear before sufficient bone formation and maturation can occur. Ideally, the degradation rate should be fast enough to allow new bone to form and mature while the material is still functioning as a scaffold [15, 27, 37]. In the present study, artificial bones composed of CPC/β-TCP composites (C30 and C50) exhibited favorable bone formation and remained between the newly formed bone and surrounding connective tissue. These findings suggest that the materials functioned as scaffolds while maintaining mechanical integrity, and that a favorable balance was achieved between bone formation and material resorption.Previous studies have employed New Zealand White rabbit femurs as experimental models. However, the developmental origins and bone formation patterns differ between long bones and craniofacial bones. Long bones of the limbs are formed through endochondral ossification from mesoderm-derived mesenchyme, whereas craniofacial bones, including the mandible, are formed through intramembranous ossification from ectodermal mesenchyme derived from the neural crest [25, 38]. In the present study, rat calvarial bone, which shares a similar developmental origin and bone formation pattern with the jawbone, was used to confirm the biocompatibility and osteoconductivity of the newly developed composite material. Based on these findings, the CPC/β-TCP composite with a ratio of 70:30 may be suitable for clinical application in the maxillofacial region, at least up to 8 weeks after implantation, in terms of bone replacement rate and mechanical strength. However, because its compressive strength remains relatively low, further investigation is required to determine whether this material can withstand vertical and horizontal alveolar bone augmentation in clinical settings. In addition, the present study has several limitations. First, although histological and histomorphometric analyses enabled evaluation of the amount and distribution of newly formed bone, the degree of mineralization of the newly formed bone and the detailed mechanisms of in vivo material resorption could not be fully assessed. Future studies incorporating additional analytical approaches, such as micro-computed tomography and immunohistochemical or other special staining techniques, may provide more comprehensive insights into bone quality and cellular-level dynamics. Second, limitations inherent to the rat calvarial defect model should be considered. The rat calvarial bone is considerably thinner than bone tissue typically encountered in clinical settings, which may have resulted in slight displacement or instability of the implanted material. In addition, variability in surgical invasiveness and intraoperative bleeding may have influenced the local healing environment and, consequently, the outcomes of bone formation and mineralization. Future studies with longer observation periods are required to evaluate the progression of bone mineralization and material resorption, as well as to elucidate in greater detail the mechanisms involved in bone formation and material degradation. Such investigations will be important for validating the applicability of the present findings to bone regenerative therapy.

## Limitation

The sample size (n = 5 per group) was determined based on the 3R principle, but statistical power to detect smaller effect sizes may have been limited. This could be one reason why no significant differences in bone formation were observed between 4 and 8 weeks in any group, despite apparent histological changes.

Furthermore, our assessment of new bone formation relied solely on H&E staining, which does not distinguish calcified bone from uncalcified osteoid. Therefore, the measured “new bone area” may include both calcified bone tissue and newly deposited but not yet calcified bone matrix (osteoid). Special staining techniques, such as Villanueva-Goldner staining (which distinguishes calcified bone as green and osteoid as red) or Masson's trichrome staining, are necessary for accurately assessing the quality and maturity of bone mineralization and represent a limitation of this study.

## Data Availability

The datasets used and/or analyzed during the current study are available from the corresponding author upon reasonable request.

## Abbreviations

CPC: Calcium phosphate cement
β-TCP: Beta-tricalcium phosphate
HA: Hydroxyapatite
α-TCP: α-Tricalcium phosphate
TTCP: Tetracalcium phosphate
DCPA: Calcium hydrogen phosphate
P: Porosity
D: Density
M: Dry weight
V: Volume
DHAp: Density of hydroxyapatite
Pr: Penetration rate

## References

[1] Stumbras A, Kuliesius P, Januzis G, Juodzbalys G. Alveolar ridge preservation after tooth extraction using different bone graft materials and autologous platelet concentrates: a systematic review. J Oral Maxillofac Res. 2019;10(1):e2. 10.5037/jomr.2019.10102. 31069040 10.5037/jomr.2019.10102 PMC6498816

[2] Zhao R, Yang R, Cooper PR, Khurshid Z, Shavandi A, Ratnayake J. Bone grafts and substitutes in dentistry: a review of current trends and developments. Molecules. 2021;26(10):3007. 10.3390/molecules26103007. 34070157 10.3390/molecules26103007 PMC8158510

[3] Kumar P, Vinitha B, Fathima G. Bone grafts in dentistry. J Pharm Bioallied Sci. 2013;5(Suppl 1):S125–7. 10.4103/0975-7406.113312. 23946565 10.4103/0975-7406.113312 PMC3722694

[4] Dimitriou R, Jones E, McGonagle D, Giannoudis PV. Bone regeneration: current concepts and future directions. BMC Med. 2011;9:66. 10.1186/1741-7015-9-66. 21627784 10.1186/1741-7015-9-66 PMC3123714

[5] Manju V, Iyer S, Menon D, Nair SV, Nair MB. Evaluation of osseointegration of staged or simultaneously placed dental implants with nanocomposite fibrous scaffolds in rabbit mandibular defect. Mater Sci Eng C Mater Biol Appl. 2019;104:109864. 10.1016/j.msec.2019.109864. 31499998 10.1016/j.msec.2019.109864

[6] Walsh WR, Vizesi F, Michael D, Auld J, Langdown A, Oliver R, et al. Beta-TCP bone graft substitutes in a bilateral rabbit tibial defect model. Biomaterials. 2008;29(3):266–71. 10.1016/j.biomaterials.2007.09.035. 18029011 10.1016/j.biomaterials.2007.09.035

[7] Titsinides S, Agrogiannis G, Karatzas T. Bone grafting materials in dentoalveolar reconstruction: a comprehensive review. Jpn Dent Sci Rev. 2019;55(1):26–32. 10.1016/j.jdsr.2018.09.003. 30733842 10.1016/j.jdsr.2018.09.003 PMC6354279

[8] Klijn RJ, Meijer GJ, Bronkhorst EM, Jansen JA. Sinus floor augmentation surgery using autologous bone grafts from various donor sites: a meta-analysis of the total bone volume. Tissue Eng Part B Rev. 2010;16(3):295–303. 10.1089/ten.TEB.2009.0558. 19958168 10.1089/ten.TEB.2009.0558

[9] Ishikawa K, Miyamoto Y, Tsuchiya A, Hayashi K, Tsuru K, Ohe G. Physical and histological comparison of hydroxyapatite, carbonate apatite, and β-tricalcium phosphate bone substitutes. Materials. 2018;11(10):1993. 10.3390/ma11101993. 30332751 10.3390/ma11101993 PMC6213161

[10] Schmidt LE, Hadad H, Vasconcelos IR, Colombo LT, da Silva RC, Santos AFP, et al. Critical defect healing assessment in rat Calvaria filled with injectable calcium phosphate cement. J Funct Biomater. 2019;10(2):21. 10.3390/jfb10020021. 31085984 10.3390/jfb10020021 PMC6616410

[11] Pommer B, Zechner W, Watzek G, Palmer GWAR. To graft or not to graft? Evidence-based guide to decision making in oral bone graft surgery. Bone Grafting. 2012;2012:1–25. 10.5772/30989.

[12] Laskus-Zakrzewska A, Kazimierczak P, Kolmas J. Porous composite granules with potential function of bone substitute and simvastatin releasing system: a preliminary study. Materials (Basel). 2021;14(17):5068. 10.3390/ma14175068. 34501158 10.3390/ma14175068 PMC8434560

[13] Ghosh R, Sarkar R. Synthesis and characterization of sintered beta-tricalcium phosphate: a comparative study on the effect of preparation route. Mater Sci Eng C Mater Biol Appl. 2016;67:345–52. 10.1016/j.msec.2016.05.029. 27287130 10.1016/j.msec.2016.05.029

[14] Yamasaki N, Hirao M, Nanno K, Sugiyasu K, Tamai N, Hashimoto N, et al. A comparative assessment of synthetic ceramic bone substitutes with different composition and microstructure in rabbit femoral condyle model. J Biomed Mater Res B Appl Biomater. 2009;91(2):788–98. 10.1002/jbm.b.31457. 19572298 10.1002/jbm.b.31457

[15] Moussa M, Carrel JP, Scherrer M, Cattani-Lorente A, Wiskott S. Durual medium-term function of a 3D printed TCP/HA structure as a new osteoconductive scaffold for vertical bone augmentation: a simulation by BMP-2 activation. Materials. 2015;8(5):2174–90. 10.3390/ma8052174. 10.1111/clr.12503 25350936

[16] Bohner M, Santoni BLG, Döbelin N. β-tricalcium phosphate for bone substitution: Synthesis and properties. Acta Biomater. 2020;1(113):23–41. 10.1016/j.actbio.2020.06.022. 10.1016/j.actbio.2020.06.022 32565369

[17] Dong J, Uemura T, Shirasaki Y, Tateishi T. Promotion of bone formation using highly pure porous beta-TCP combined with bone marrow-derived osteoprogenitor cells. Biomaterials. 2002;23(23):4493–502. 10.1016/s0142-9612(02)00193-x. 12322969 10.1016/s0142-9612(02)00193-x

[18] Ogose A, Hotta T, Kawashima H, Kondo N, Gu W, Kamura T, et al. Comparison of hydroxyapatite and beta tricalcium phosphate as bone substitutes after excision of bone tumors. J Biomed Mater Res B Appl Biomater. 2005;72(1):94–101. 10.1002/jbm.b.30136. 15376187 10.1002/jbm.b.30136

[19] Anna D, Montserrat E, Maria G. Chapter 5-Synthetic bone graft substitutes: calcium-based biomaterials. In: Dental Implantss and Bone Grafts Materials and Biological Issues. Cambridge: Woodhead Publishing; 2020. p. 125–57. 10.1016/B978-0-08-102478-2.00006-4.

[20] Ginebra MP, Canal C, Espanol M, Pastorino D, Montufar EB. Calcium phosphate cements as drug delivery materials. Adv Drug Deliv Rev. 2012;64(12):1090–110. 10.1016/j.addr.2012.01.008. 22310160 10.1016/j.addr.2012.01.008

[21] Chow LC. Next generation calcium phosphate-based biomaterials. Dent Mater J. 2009;28(1):1–10. 10.4012/dmj.28.1. 19280963 10.4012/dmj.28.1 PMC2721275

[22] Lodoso-Torrecilla I, van den Beucken JJJP, Jansen JA. Calcium phosphate cements: Optimization toward biodegradability. Acta Biomater. 2021;119:1–12. 10.1016/j.actbio.2020.10.013. 33065287 10.1016/j.actbio.2020.10.013

[23] Sawamura T, Mizutani Y, Okuyama M, Obata A, Kasuga T. Compressive strength of calcium phosphate cements prepared using different initial setting temperatures. J Ceram Soc Japan. 2015;123:59–61.

[24] Tanaka H, Yamada S, Aizawa H, Hayashi K, Shimane T, Karasawa I, et al. Mechanical properties and histological evaluation of bone grafting materials containing different ratios of calcium phosphate cement and porous β-tricalcium phosphate granules. Shinshu Med J. 2018;66:139–50.

[25] Vaquette C, Mitchell J, Fernandez-Medina T, Kumar S, Ivanovski S. Resorbable additively manufactured scaffold imparts dimensional stability to extraskeletally regenerated bone. Biomaterials. 2021;269:120671. 10.1016/j.biomaterials.2021.120671. 33493771 10.1016/j.biomaterials.2021.120671

[26] Bohner M, Gbureck U, Barralet JE. Technological issues for the development of more efficient calcium phosphate bone cements: a critical assessment. Biomaterials. 2005;26(33):6423–9. 10.1016/j.biomaterials.2005.03.049. 15964620 10.1016/j.biomaterials.2005.03.049

[27] Hayashi K, Kishida R, Tsuchiya A, Ishikawa K. Honeycomb blocks composed of carbonate apatite, β-tricalcium phosphate, and hydroxyapatite for bone regeneration: effects of composition on biological responses. Mater Today Bio. 2019;4:100031. 10.1016/j.mtbio.2019.100031. 32159156 10.1016/j.mtbio.2019.100031 PMC7061555

[28] Mastrogiacomo M, Scaglione S, Martinetti R, Dolcini L, Beltrame F, Cancedda R, et al. Role of scaffold internal structure on in vivo bone formation in macroporous calcium phosphate bioceramics. Biomaterials. 2006;27(17):3230–7. 10.1016/j.biomaterials.2006.01.031. 16488007 10.1016/j.biomaterials.2006.01.031

[29] Kuboki Y, Takita H, Kobayashi D, Tsuruga E, Inoue M, Murata M, et al. BMP-induced osteogenesis on the surface of hydroxyapatite with geometrically feasible and nonfeasible structures: topology of osteogenesis. J Biomed Mater Res. 1998;39(2):190–9. 10.1002/(sici)1097-4636(199802)39:2<190::aid-jbm4>3.0.co;2-k. 9457547 10.1002/(sici)1097-4636(199802)39:2<190::aid-jbm4>3.0.co;2-k

[30] Karageorgiou V, Kaplan D. Porosity of 3D biomaterial scaffolds and osteogenesis. Biomaterials. 2005;26(27):5474–91. 10.1016/j.biomaterials.2005.02.002. 15860204 10.1016/j.biomaterials.2005.02.002

[31] Albrektsson T, Johansson C. Osteoinduction, osteoconduction and osseointegration. Eur Spine J. 2001;10(2):S96-101. 10.1007/s005860100282. 11716023 10.1007/s005860100282 PMC3611551

[32] Watanabe T, Takabatake K, Tsujigiwa H, Watanabe S, Nakagiri R, Nakano K, et al. Effect of honeycomb β-TCP geometrical structure on bone tissue regeneration in skull defect. Materials. 2020;13(21):4761. 10.3390/ma13214761. 33113818 10.3390/ma13214761 PMC7663559

[33] Van Bael S, Chai YC, Truscello S, Moesen M, Kerckhofs G, Van Oosterwyck H, et al. The effect of pore geometry on the in vitro biological behavior of human periosteum-derived cells seeded on selective laser-melted Ti6Al4V bone scaffolds. Acta Biomater. 2012;8(7):2824–34. 10.1016/j.actbio.2012.04.001. 22487930 10.1016/j.actbio.2012.04.001

[34] Fernández RF, Bucchi C, Navarro P, Beltrán V, Borie E. Bone grafts utilized in dentistry: an analysis of patients’ preferences. BMC Med Ethics. 2015;16(1):71. 10.1186/s12910-015-0044-6. 26486125 10.1186/s12910-015-0044-6 PMC4618514

[35] Araújo MG, Lindhe J. Dimensional ridge alterations following tooth extraction. An experimental study in the dog. J Clin Periodontol. 2005;32(2):212–8. 10.1111/j.1600-051X.2005.00642.x. 15691354 10.1111/j.1600-051X.2005.00642.x

[36] Peitsinis PR, Blouchou A, Chatzopoulos GS, Vouros ID. Optimizing implant placement timing and loading protocols for successful functional and esthetic outcomes: a narrative literature review. J Clin Med. 2025;14(5):1442. 10.3390/jcm14051442. 40094901 10.3390/jcm14051442 PMC11900159

[37] Rojbani H, Nyan M, Ohya K, Kasugai S. Evaluation of the osteoconductivity of α-tricalcium phosphate, β-tricalcium phosphate, and hydroxyapatite combined with or without simvastatin in rat calvarial defect. J Biomed Mater Res A. 2011;98(4):488–98. 10.1002/jbm.a.33117. 21681941 10.1002/jbm.a.33117

[38] van den Bos T, Speijer D, Bank RA, Brömme D, Everts V. Differences in matrix composition between calvaria and long bone in mice suggest differences in biomechanical properties and resorption: special emphasis on collagen. Bone. 2008;43(3):459–68. 10.1016/j.bone.2008.05.009. 18583211 10.1016/j.bone.2008.05.009

