# Correction to: Evaluation of the composite materials mixed with calcium phosphate cement and β-tricalcium phosphate granules

**DOI:** 10.1186/s40729-026-00677-3

**Published:** 2026-04-07

**Authors:** Eri Yumoto, Hironori Sakai, Yang Liu, Jianping Liu, Ryo Okaniwa, Ryo Kajihara, Hirokazu Tanaka, Hiroshi Kurita

**Affiliations:** 1 Department of Dentistry and Oral Surgery, Shinshu University School of Medicine, 3-1-1, Asahi, Matsumoto, 390-8621 Japan; 2 Department of Oral and Maxillofacial Surgery, School and Hospital of Stomatology, Hebei Technology Innovation Center of Oral Health, Hebei Medical University, Shijiazhuang, 050017 Hebei Province China

**Correction to: International Journal of Implant Dentistry (2026) 12:8** 10.1186/s40729-026-00670-w

The original publication of this article contained an error in the setting time of Biopex®-R. The incorrect and correct information is shown in this correction article. This does not impact the results nor conclusions of the article. The original article has been updated.

**Incorrect**

- Although the previously used CPC (Cerapaste®) typically required 5–20 min to set, Biopex®-R reportedly sets in approximately **7** min according to manufacturer information.

**Correct**

- Although the previously used CPC (Cerapaste®) typically required 5–20 min to set, Biopex®-R reportedly sets in approximately **4** min according to manufacturer information.

